# A platform for rapid generation of single and multiplexed reporters in human iPSC lines

**DOI:** 10.1038/srep09205

**Published:** 2015-03-17

**Authors:** Ying Pei, Guadalupe Sierra, Renuka Sivapatham, Andrzej Swistowski, Mahendra S. Rao, Xianmin Zeng

**Affiliations:** 1 Buck Institute for Age Research, Novato, CA; 2 XCell Science, Novato, CA; 3 NxCell Inc, Novato, CA

## Abstract

Induced pluripotent stem cells (iPSC) are important tools for drug discovery assays and toxicology screens. In this manuscript, we design high efficiency TALEN and ZFN to target two safe harbor sites on chromosome 13 and 19 in a widely available and well-characterized integration-free iPSC line. We show that these sites can be targeted in multiple iPSC lines to generate reporter systems while retaining pluripotent characteristics. We extend this concept to making lineage reporters using a C-terminal targeting strategy to endogenous genes that express in a lineage-specific fashion. Furthermore, we demonstrate that we can develop a master cell line strategy and then use a Cre-recombinase induced cassette exchange strategy to rapidly exchange reporter cassettes to develop new reporter lines in the same isogenic background at high efficiency. Equally important we show that this recombination strategy allows targeting at progenitor cell stages, further increasing the utility of the platform system. The results in concert provide a novel platform for rapidly developing custom single or dual reporter systems for screening assays.

Induced pluripotent stem cells (iPSC) are rapidly becoming the mainstay of *in vitro* human cell-based assays for both toxicology and drug discovery. This has been possible due to a slew of advances in the field, which include techniques for high efficiency homologous recombination using transcription activator-like effector nuclease (TALEN), zinc finger nucleases (ZFN) or clustered regularly interspaced short palindromic repeats (CRISPRs)/cas9 system^1^,^2^,^3^,^4^, and the ability to make integration-free iPSC cost effectively from normal individuals and patients with monogenic and polygenic diseases. Combined with advances in differentiating iPSC into multiple cell types, this allows the same signaling pathways or the same mutation to be assessed in a common allelic background. The power of this approach has been demonstrated by multiple groups using human cells rather than the standard xeno models used in the past^5^,^6^,^7^,^8^. Other groups have used iPSC-based models to identify patients who might adversely respond to an approved drug therapy or discover new drugs to treat a disease^9^,^10^,^11^,^12^,^13^.

Although these efforts clearly demonstrate the utility of using iPSC-derived cells for screening and toxicology assays, several issues have constrained the widespread use of such cells. Perhaps the biggest issues are the time periods required to differentiate iPSC into an appropriate phenotype, the purity of the differentiated cells, and the consistency of the differentiation process. Further constraints include the lack of isogenic lines to control for allelic variability, the difficulty in generating reporter systems, and the time required to select stable subclones for assays^14^,^15^,^16^,^17^,^18^. Several groups have begun to develop techniques to address these problems. For example researchers have shown that ZFN, TALEN and CRISPRs/cas9 systems provide efficient gene targeting technologies and allow one to develop safe harbor or lineage specific reporter system^19^,^20^,^21^,^22^. Our laboratories have also shown that it is possible to make GFP and luciferase reporter lines using a standardized targeting system for safe harbor sites where expression is not silenced during differentiation^21^. This has extended the range of targets for which iPSC-derived cells can be used.

To further extend the utility of iPSC and somatic cells for such assays we designed high efficiency TALEN to target safe harbor sites on chromosomes 19 (Chr. 19) and 13 (Chr. 13) to generate monoallelic and biallelic reporters. We further extended this targeting strategy to making lineage-specific reporters by targeting a Nanoluc-Halotag construct to the C-terminal of the GFAP and MAP2 genes. We demonstrated that these subclones recapitulate with high fidelity the lineage specific expression of the endogenous gene, and allowed detailed mapping of the differentiation processes. To enhance the flexibility of the targeting strategy, we developed a master cell line where different constructs can be rapidly replaced at the same site using the Cre-Lox system to generate multiple novel subclones with high efficiency. This enhanced efficiency permits cassette exchange even in cells that are further differentiated. We showed that neural stem cells could be readily targeted. Overall our results confirm that we have a novel platform for rapidly developing single and multiplexed reporter lines, and the high efficiency constructs can be used to make similar lines in other normal and patient specific lines.

## Results

### Efficient targeting of Chr. 19 and Chr. 13 safe harbor loci in multiple lines with multiple constructs

**Figure 1:**
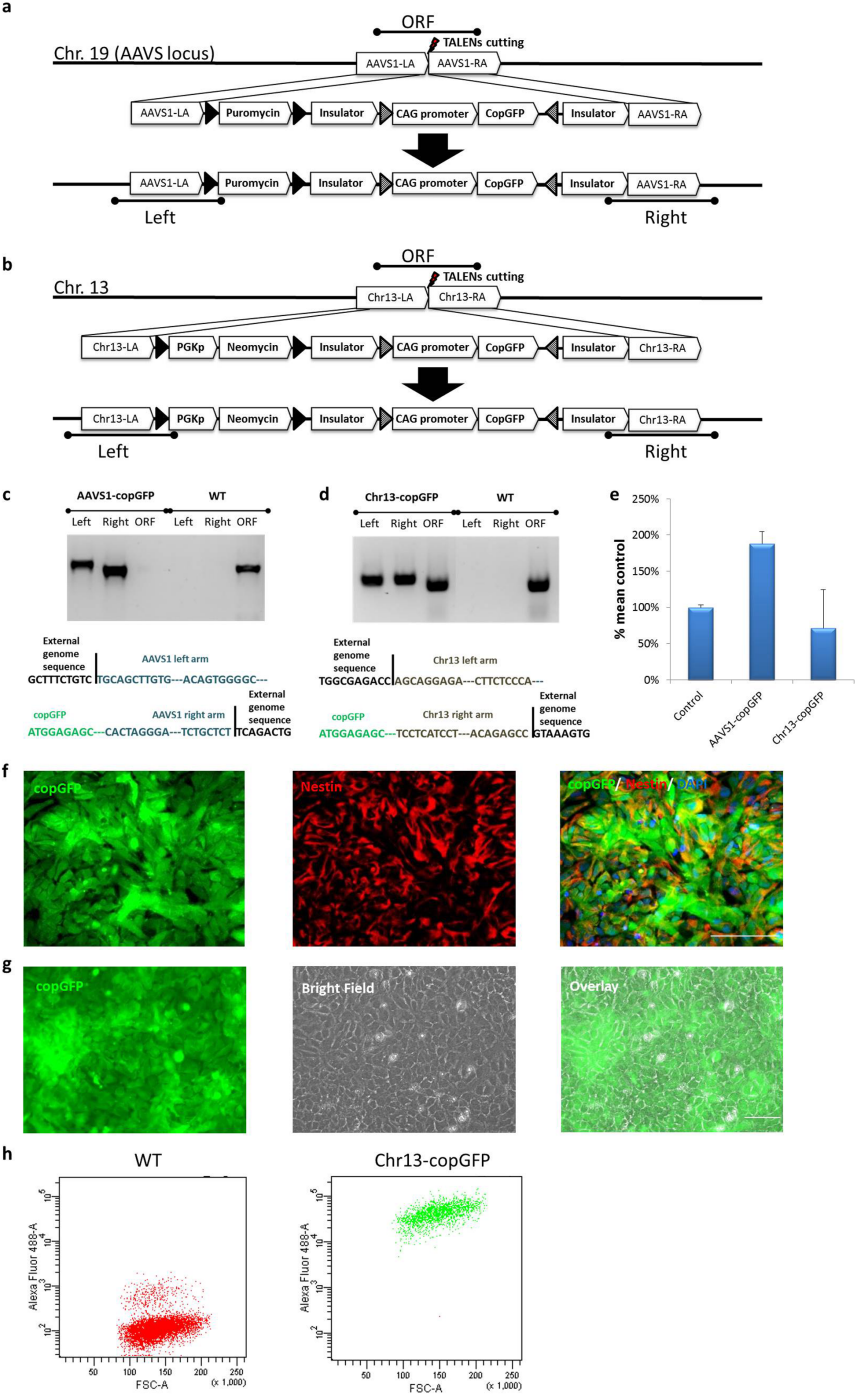
Efficient targeting of Chr. 19 and Chr. 13 safe harbor loci. The experimental strategy of generating AAVS1-copGFP (a) and Chr13-copGFP (b) iPSC lines. Solid black triangles represent the loxP sites and triangles filled with diagonal lines represent Lox sites for RMCE. Testing primer sets for “Left” (Left arm integration test), “Right” (Right arm integration test) and “ORF” (WT ORF test) were also illustrated. (c) Validation of AAVS1-copGFP heterozyte and homozygote clones by junction PCR (upper) and sequencing (lower). (d) Validation of Chr13-copGFP heterozygote clone by junction PCR (upper) and sequencing (lower). (e) Quantification of donor vector copy number via qPCR. Data were presented as %mean control using the previously validated single copy line “CAG-GFP-Chr19” as the 100% control. (f) The copGFP reporter gene in AAVS1-copGFP line was not silenced while differentiated into NSC. Nestin (red) antibody was used to label NSC. Scale bar is 100 μm. (g) The copGFP reporter gene in Chr13-copGFP line was not silenced while differentiated into NSC. Scale bar is 100 μm. (h) FACS analysis of GFP-positive cells in a representative culture (Chr13-copGFP NSC) (right panel) and the non-labeled WT control NSC (left panel).

We tested two safe harbor sites for efficient generation of knock-in (KI) reporters in iPSC via TALEN and ZFN-mediated homologous recombination^21^,^23^. The data presented here were by TALEN. The first site we targeted was the commonly employed AAVS1 site on Chr.19 and the second site we chose was the site on Chr.13. These sites have previously been shown not to be silenced^21^,^23^. Different promoters were evaluated and the CMV early enhancer/chicken beta actin (CAG) promoter appeared the most stable and was used for subsequent experiments (data not shown). Additionally, two different reporters were evaluated: Nanoluc (luciferase) for quantitation and sensitivity, and copGFP for its fluorescence intensity and stability, as well as our ability to get license to distribute the cell lines. Both reporters worked efficiently and a subset of the data is shown in Fig. 1 and Sup. Fig 1.

The constructs and the schemas of generating KI iPSC lines at the two safe harbor sites with the reporter (e.g. copGFP) driven by the constitutively active CAG promoter are illustrated in Fig. 1a–b. A well-characterized integration-free iPSC line, XCL1, was used as the parental line for all gene-targeting work described in this study unless specified otherwise. We first targeted the Chr. 19 site and after drug selection on 2 × 10^6^ post nucleofection cells we observed ~ 80 puromycin resistant colonies. From these drug resistant colonies, we analyzed 37 colonies for AAVS1-copGFP line by PCR and single cell colony cloning. Of them 12 clones were targeted on one allele and 25 were targeted to both alleles. Similar targeting efficacy was observed for the Chr. 13 site. A representative example of a monoallelic (heterozygote) and biallelic (homozygote) was shown in Fig, 1c–d (homozygotes for the AAVS1-copGFP line and heterozygotes for the Chr13-copGFP line). Further sequencing of the PCR products confirmed the successful integration of donor constructs into appropriate genome loci (Fig. 1c and 1d). The copy number of the donor vector in both AAVS1-copGFP (homozygote) and Chr13-copGFP (heterozygote) lines was validated by qPCR (Fig. 1e) using the CAG-GFP-Chr19 iPSC line as a positive control (the copy number of this line was previously verified by Southern blot^21^). We showed that AAVS1-copGFP (homozygote) had two copies of the donor vector while the Chr13-copGFP (heterozygote) line had one copy of the donor vector. Quantitative expression of the insert allowed us to distinguish between a single copy insert and a multiple insertion. Since the levels we saw were consistent with a single or double copy insert, it was unlikely that a full copy insertion at an ectopic site. Combined with our PCR showing the correct gDNA recombination (1c & 1d), we concluded that our donor vector was successfully integrated into the right locus of the genome.

We then validated the reporter lines engineered by each of the safe harbor integration strategies. Genomic stability of a representative line (the AAVS1-copGFP line) was shown in Sup Fig. 1a. When directly differentiated toward the neural lineage, the copGFP reporter in this KI line was not silenced as evidenced by continuous expression of GFP in nestin-positive neural stem cell (NSC) (Fig. 1f). No gene silencing was observed during random differentiation via embryoid body formation as cells of the three germ layers differentiated from the AAVS1-copGFP line remained GFP-positive (Sup Fig. 1b). In addition, copGFP reporter in the Chr13-copGFP line was tested and no gene silencing was detected in the derived NSC (Fig. 1g & 1h).

To confirm that our safe harbor KI approach can be generalized, we created similar reporters in another well-characterized integration-free line, XCL5 (NCRM5). As an example, we generated a Chr13-Nanoluc-halotag line, similar to the Chr13-copGFP line in which a Nanoluc/HaloTag reporter was used instead of copGFP (construct not shown). We differentiated the line to a pure population of neurons or astrocytes via directed differentiation and confirmed no gene silencing in these lineages (Sup. Fig.1c). Taken together, targeting at the safe harbor loci was both reliable and efficient. The cell lines obtained were stable, karyotypically normal and the reporters did not silence on random or directed differentiations. In addition, both sites could be targeted simultaneously, and both monoallelic and biallelic subclones could be identified.

### Rapid exchanging of reporter cassettes in safe harbors in iPSC and progenitor cells using a master cell line strategy

**Figure 2:**
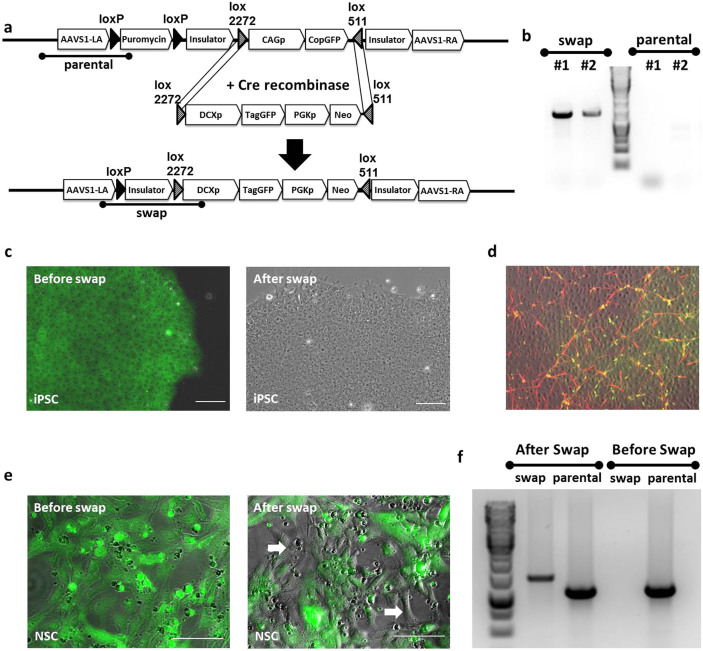
Rapid exchanging of reporter cassettes in safe harbors in iPSC and progenitor cells using a master cell line strategy. (a) The experimental strategy of quick and efficient generation of a reporter line via RMCE strategy. The master line, AAVS1-copGFP, was co-transfected with the Cre expression vector and the targeting plasmid DCXp-TagGFP carrying a *lox*2272-DCXpromoter-TagGFP-PGK-Neo-*lox*511 cassette for RMCE with the master line. Cells with successfully targeted recombination were neomycin resistant and expressed TagGFP under the endogenous promoter of DCX instead of the constitutive CAG promoter which is seen in the master line. Testing primers, “swap” (indicating the successful RMCE event) and “parental” (detecting the parental gene which has no swap event) were also illustrated. Solid black triangles represent the LoxP sites and triangles filled with diagonal lines represent Lox sites for RMCE. (b) PCR verification of the selected non-fluorescent colonies. No “parental” PCR products were detected in any of the selected colonies. (c) After Neomycin selection, colonies with no copGFP signal were selected under fluorescent microscope. Overlaying fluorescence channel with the bright field, the cells before swap were all green fluorescent (left). After correct swap, the CAG promoter driving copGFP was replaced by DCX promoter driving TagGFP whose expression is off at the iPSC stage, and cells were no longer fluorescent (right). (d) Neuronal differentiation was induced on iPSC selected above and immunostaining showed positive co-localization of DCX antibody (red) and TagGFP (green), suggesting that the TagGFP is only expressed when the DCX gene is turned on. (e) RMCE strategy was also tested in the progenitor stage (NSC). AAVS1-copGFP master line NSC were co-transfected with Cre expressing vector and the targeting plasmid DCXp-TagGFP as described in (a). Before transfection, all AAVS1-copGFP NSC were green fluorescent (left). After 5 days post transfection, cells lost green fluorescence were detected under microscope (spots pointed by arrows in the right graph). Images shown here are bright field superimposed with fluorescence channel. (f) PCR verification of successful swapping event happened in NSC. Only “parental” PCR band was detectable in NSC before transfection. After RMCE, both “swap” and “parental” PCR products were detected in the mixed culture. Scale bar is 100 μm.

Although ZFN and TALEN increased targeting efficiency several-fold compared to the traditional gene targeting methods, we were unsure if the efficiency would be high enough that we could target non-pluripotent cells. This may be important when the differentiation process is very long or genes have toxic effects at some stages. We therefore redesigned our safe harbor site targeting strategy by utilizing constructs with multiple Lox sites, which allowed us to easily replace one reporter or promoter with another by Cre-recombinase mediated cassette exchange (RMCE). An example of such a vector design is illustrated in Fig. 2a. In this construct, the CAG promoter driving the copGFP reporter cassette was inserted between lox2272 and lox511 sites with the appropriate orientation for RMCE. In addition, a puromycin resistant gene flanked by two different loxP sites was inserted at the endogenous promoter of AAVS1. Two insulator sites were also added in this line to prevent copGFP silencing (Fig. 2a). To generate new reporter lines, daughter constructs containing any gene-specific or ubiquitous promoter driving a reporter gene can be inserted between a lox2272 and a lox511 site, and drug selection and loss of the previous insert can be used to identify appropriate clones.

We tested this strategy by replacing GFP driven by the ubiquitous CAG promoter in the AAVS1-copGFP line with a promoter-reporter construct using the neuronal lineage-specific promoter doublecortin (DCX) driving TagGFP (Fig. 2) or Nanoluc (data not shown). In the DCX daughter construct, DCXp-TagGFP, a DCX promoter driving TagGFP together with a PGK promoter driving Neomycin resistant gene was cloned between lox2272 and lox511 sites (Fig. 2a). In order to induce the RMCE, DCXp-TagGFP construct was co-transfected with a plasmid expressing Cre recombinase by the PGK promoter into established AAVS1-copGFP (a homozygote clone) iPSC line. After RCME, colonies that had lost green fluorescence were identified and picked for PCR verifications. Using primers designed specifically for targeting the “parental” and “swap” sequences, we were able to identify iPSC clones where cassette exchange had been successful (Fig. 2b). Before cassette exchange, the master iPSC line AAVS1-copGFP constitutively expresses green fluorescence and is puromycin resistant. In the presence of Cre recombinase and DCX daughter construct, the puromycin gene is deleted via Cre-loxP mediated recombination, and “CAGp-copGFP” is replaced by the “DCXp-TagGFP-PGKp-Neo” cassette. Thus the new reporter line, named DCXp-TagGFP, is not puromycin but neomycin resistant and is not fluorescent at the iPSC stage (Fig. 2c).

To confirm functionally appropriate expression in the DCXp-TagGFP reporter line created by cassette exchange, we used a directed differentiation protocol to induce neuronal differentiation as previously described^24^. As the cells differentiated toward the neuronal lineage, GFP-positive cells appeared (Fig. 2d). Immunocytochemistry staining 6 days after differentiation confirmed that all green cells were specifically located with DCX antibody positive neurons (Fig. 2d), validating the specificity of the DCXp-TagGFP reporter line.

To further confirm the utility of the master iPSC line strategy, we tested whether the RMCE can be extended to intermediate/progenitor stage cells as well. Given our extensive experience in neural lineage, we tested the RMCE at the NSC stage as a proof-of-concept. We first derived NSC from the AAVS1-copGFP iPSC line, which maintained strong green fluorescence through differentiation (Fig. 1e & 2e). Following the same RMCE procedures as described for the iPSC (Fig. 2a), DCXp-TagGFP daughter construct and Cre-expressing plasmid were co-transfected into AAVS1-copGFP NSC. No drug selection was used to enrich cells with successful RMCE, since our goal for this experiment was not to isolate single clones of NSC with correct cassette exchange. Instead, we analyzed the entire cell population and identified cells losing green fluorescence post transfection by fluorescence microscopy, indicating the successful event of RMCE (Fig. 2e). Junction PCR was used to confirm that cassette exchange was indeed correctly induced in a subset of the NSC (Fig. 2f). Overall these results showed that isogenic subclones can be rapidly generated at multiple stages of differentiation, which should allow expression of deleterious genes at specific stages of development.

### Generation and lineage-specific expression of KI reporters

**Figure 3:**
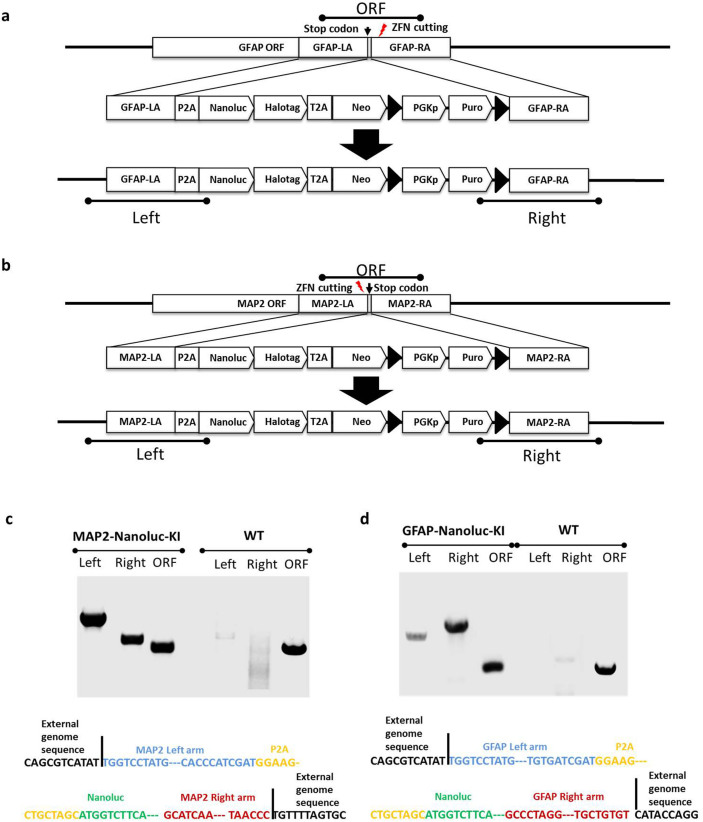
Generation of knock-in lines at lineage specific genes. (a) Experimental strategy of generating MAP2-Nanoluc-KI. The designed ZFNs cut at the C-term of MAP2 gene before the stop codon and the left and right arms of MAP2 for homologous recombination were designed to be ~ 1 kb located before and after the stop codon, respectively. Testing primer sets for “Left” (MAP2 Left arm integration test), “Right” (MAP2 Right arm integration test) and “ORF” (WT MAP2 ORF test) were also illustrated. (b) Experimental strategy of generating GFAP-Nanoluc-KI. The designed ZFNs cut after the stop codon of GFAP ORF. The left and right arms of GFAP for homologous recombination were designed to be ~ 1 kb located before and after the stop codon, respectively. Testing primer sets for “Left” (GFAP Left arm integration test), “Right” (GFAP Right arm integration test) and “ORF” (WT GFAP ORF test) were also illustrated. (c) Validation of MAP2-Nanoluc-KI clone by junction PCR (upper) and sequencing (lower). (d) Validation of GFAP-Nanoluc-KI clone by junction PCR (upper) and sequencing (lower).

Although lineage-specific constructs for some genes are available and some fragments are sufficiently small that they could be targeted to the safe harbor loci (see above DCXp-copGFP reporter), we wished to develop a KI strategy in genes that are expressed in specific lineages to allow the development of assays to identify regulators of development. Given our interest in neural development we chose to knock-in a Nanoluc-HaloTag construct (Fig. 3) into the endogenous MAP2 locus, and the same reporter construct in endogenous GFAP allele. We elected to make monallelic lines and target the 3′ prime end of the gene to allow expression of normal levels of the endogenous gene. Both KI reporter lines were made in the XCL1 iPSC line to show that isogenic subclones could be obtained, but the same construct has been used in other NCRM lines as well. Specifically, ZFN pairs targeting the C-term of GFAP or MAP2 genes were designed and optimized. One pair cutting at ~ 130 bp after the stop codon of GFAP ORF and ZFNs targeting ~ 90 bp before the stop codon of MAP2 gene were selected in this study (Fig. 3a & 3b). A donor vector consisting of a reporter cassette of a P2A peptide, a Nanoluc luciferase gene fused with a HaloTag, followed by a neomycin resistance gene was designed to be in frame with the C-terminal of the targeted genes (Fig. 3a & 3b). After co-transfection with the donor vector and mRNA of the respective ZFN pair on 2 × 10^6^ cells and drug selection, we observed ~ 60 puromycin resistant colonies. Thirty-six drug resistant colonies from each line were picked for further analysis. Successful insertion of the reporter genes to either GFAP or MAP2 gene was confirmed by both PCR and sequencing analyses for 33 GFAP and 4 MAP2 clones (Fig. 3c & 3d). Four clones of each reporter lines were selected and verified to be heterozygotes (Fig. 3c & 3d). One of each validated GFAP-Nanoluc-KI and MAP2-Nanoluc-KI clone was chosen for further analysis as described below.

**Figure 4:**
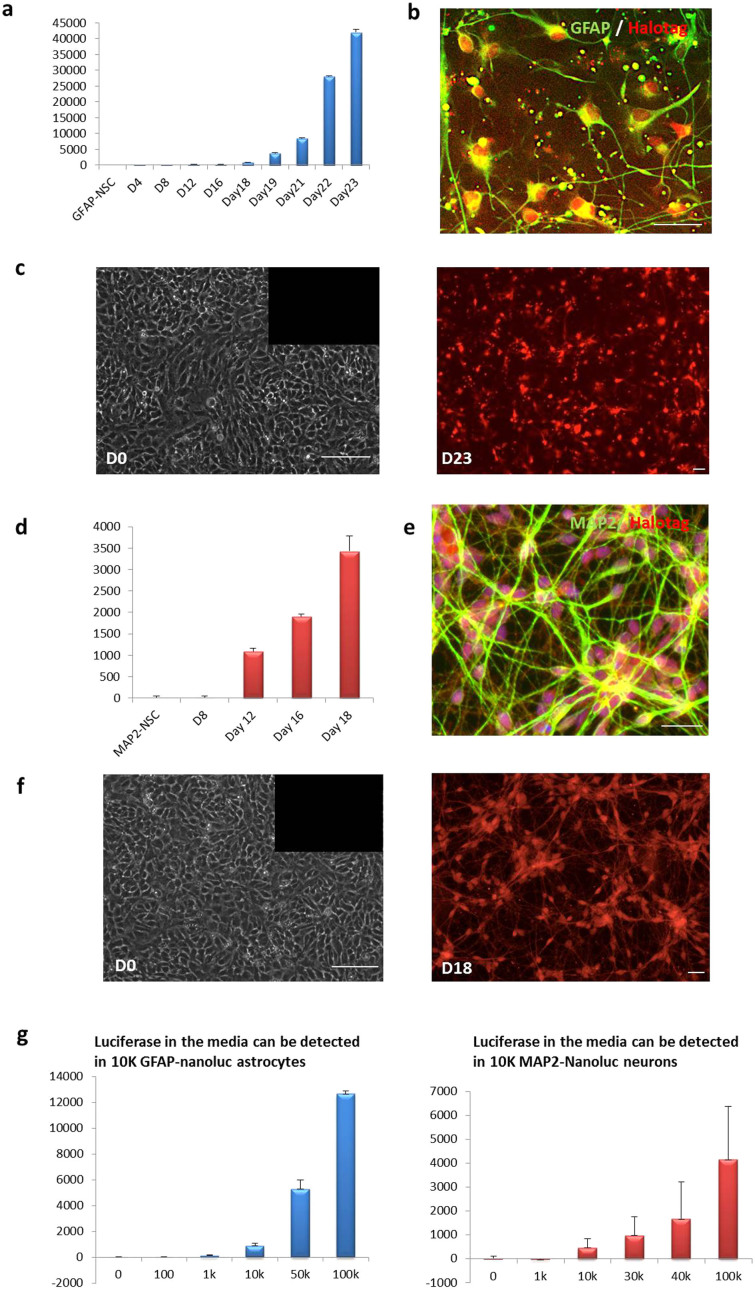
Functional validation of lineage-specific expression. (a) An increase of luciferase level in culture media was detected in the GFAP-Nanoluc-KI cell lines during directed differentiation into astrocytes. Luciferase levels shown in the bar graph were normalized by the basal level detected at day 0 in GFAP-Nanoluc-KI NSC. (b) Immunostaining showed excellent co-localization of HaloTag (red) and GFAP (green) antibodies in the GFAP-Nanoluc-KI astrocytes (D23 post differentiation). (c) Live staining of HaloTag (red) in the GFAP-Nanoluc-KI cell line before (left) and after (right) directed differentiation into astrocytes. (d) An increase of luciferase level in culture media was detected in the MAP2-Nanoluc-KI cell lines during directed differentiation into neurons. Luciferase levels shown in the bar graph were normalized by the basal level detected at day 0 in the MAP2-Nanoluc-KI NSC. (e) Immunostaining showed excellent co-localization of HaloTag (red) and MAP2 (green) antibodies in the MAP2-Nanoluc-KI neurons (D18 post differentiation). (f) Live staining of HaloTag (red) in the MAP2-Nanoluc-KI cell line before (left) and after (right) directed differentiation into neurons. (g) A series dilution of GFAP-Nanoluc-KI (left) and MAP2-Nanoluc-KI (right) iPSC were plated and luciferase level from the media was tested. The minimum cell amount needed was 10 K for detectable luciferase level of both GFAP-Nanoluc-KI and MAP2-Nanoluc-KI iPSC lines. Scale Bars shown in this Fig are all 100 μm.

We first confirmed the positive expression of pluripotency markers and a normal karyotype in prolonged culture (Sup. Fig. 2) of both GFAP-Nanoluc-KI and MAP2-Nanoluc-KI iPSC lines. We next induced neural differentiation via NSC formation from these two iPSC lines and tracked the expression of the reporter genes, Nanoluc and HaloTag, during lineage-specific differentiation (Fig. 4). As expected, no luciferase signal was detected in GFAP-Nanoluc-KI or MAP2-Nanoluc-KI lines or NSC derived from them (Fig. 4a, 4c, 4d & 4f).

For GFAP-Nanoluc-KI NSC, we monitored the expression of luciferase and HaloTag during astrocyte differentiation using a well-established protocol by our laboratory^5^. Starting from day 18 after the NSC stage, the luminescence intensity increased gradually as the cells differentiated to astrocytes (Fig. 4a). In order to visualize expression of the reporter gene during differentiation, ligand that covalently binds to HaloTag was used to label live GFAP-Nanoluc-KI cells at different time points during astrocyte differentiation. HaloTag-labeled fluorescent cells were only observed after the differentiation, further confirming that the reporters were turned on specifically by the GFAP promoter as the cells differentiated into astrocytes (Fig. 4c). We then tested the differentiated GFAP-Nanoluc-KI cells (D23 post differentiation) by immunostaining and found co-localization of GFAP and HaloTag antibodies in nearly 100% of the cells, indicating that the reporter genes in GFAP-Nanoluc-KI only turned on in the GFAP-positive cells (Fig 4b).

Using a similar strategy, we monitored the expression of Nanoluc and HaloTag reporter genes in the MAP2-Nanoluc-KI cells during neuronal differentiation. We used a previously reported 2-week differentiation protocol to generate a pure population of mixed neurons from the NSC^25^. No detectable luminescence was observed until 12 days post differentiation (Fig. 4d). No fluorescence was detected from HaloTag expression in MAP2 NSC prior to differentiation (Fig. 4f). Expression of HaloTag was observed as more and more cells differentiated into neurons (Fig. 4e). Importantly, HaloTag antibody only stained the MAP2-positive neurons, indicating the specific expression of the reporter from the endogenous MAP2 promoter. These results signify that a single luciferase or HaloTag gene is sufficiently sensitive to allow for live tracking of differentiation events.

To determine the minimal numbers of cells required for detectable level of Nanoluc, we measured the luminescence level of both GFAP-Nanoluc-KI astrocytes and MAP2-Nanoluc-KI neurons at different cell densities (Fig. 4g). Less than 1 × 10^4^ cells were required for either GFAP-Nanoluc-KI astrocytes or MAP2-Nanoluc-KI neurons to be detected by luminescence in a 96-well format. This result suggested that the GFAP-Nanoluc-KI and MAP2-Nanoluc-KI reporters can be used to effectively and accurately track astrocytes or neurons during differentiation with small numbers of cells and in a high-throughput manner.

To further demonstrate that clones with multiple reporters can be made rapidly, we generated a stock of NSC from the MAP2-Nanoluc-KI subclone and targeted the safe harbor locus at the NSC stage. As expected, the targeted clone (the MAP2-Nanoluc-KI line) could be re-targeted and a dual reporter line could be readily generated (data not shown). Even higher efficiency was obtained when the clone was targeted at the iPSC stage, which was comparable to those seen in an untargeted line.

Although we did not observe any effects on adjacent genes or overall gene expression in the defined safe harbor sites^26^, we had not however previously reported on the MAP2 and GFAP locus. Examination of these two lines by whole genome profiling did not show any alterations in gene expression at the iPSC stage (data not shown). However since these genes are only expressed in specific lineages we differentiated the lines into astrocytes and neurons respectively. We then specifically examined gene expression on Chr. 17 (where GFAP gene locates) and Chr. 2 (where MAP2 gene locates) and found no significant difference (Data included in a Sup table which is available upon request). We then identified the genes adjacent to GFAP and MAP2 and looked for significant changes and no dramatic changes were seen (Sup Table 1).

Overall, our results suggest that with a combination of safe harbor gene editing, cassette exchange tools, and the identification of lineage specific gene loci, one can rapidly develop single and multiplexed reporters that provide investigators to develop a repertoire of assays using reporters appropriate for their particular need.

## Discussion

**Figure 5:**
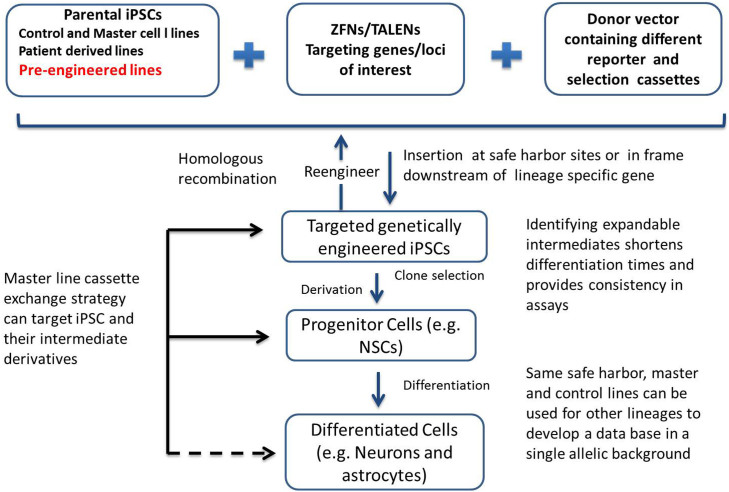
Summary of different approaches to generate reporter lines in safe harbors and in endogenous lineage-specific genes. Genetic modification techniques (ZFNs or TALEN) were used in combination with carefully designed donor vectors in this study to target and modify genes/loci-of-interest in selected parental iPSC lines. The parental iPSC can be well-established control lines, patient-derived lines, pre-engineered lines or master lines for the quick swapping strategy. Depending on the donor vectors and targeting genes/loci, parental iPSC can be engineered or re-engineered into different lines expressing either constitutively active reporter genes at the safe harbors or reporter genes that are in frame downstream of lineage-specific genes. These targeted genetically engineered iPSC can be derived into progenitor cells and further differentiated into different cell types for numerous screening purposes. Additionally, we illustrated a master line cassette exchange strategy which gave us the opportunity to quickly and efficiently generate different reporter lines at the safe harbor sites. Using this strategy, we showed successful targeting to both iPSC and the progenitor cells (solid arrows). Potentially, this strategy can also be applied directly to the differentiated cells (dotted arrow).

One important feature of iPSC is that they can be engineered in multiple ways to be used for screenings, for developing therapeutic purposes, or for investigating disease mechanisms or processes. For instance, iPSC can be engineered to create ubiquitous reporters to develop enhanced assays, lineage-specific reporters to allow for stage-specific screening, or pathway and organelle reporters to allow for focused screening. To enable these strategies we have developed a novel iPSC-based platform for rapid development of reporter lines, which utilizes safe harbor sites, a master cell line strategy combined with CRE-mediated recombination, and linage-specific gene expression. Vector constructs used to generate these lines have been carefully designed, and promoters and reporters carefully chosen so that they can be multiplexed to provide ratio-metric measurements and quantitative analysis, or be used to monitor lineage-specific differentiation *in vitro* *and in vivo*. The methodology has been optimized to ensure that similar reporters can be made in multiple lines with similar efficiency and consistent readout. Furthermore, the assays can be multiplexed in the same isogenic line. Fig. 5 summarizes these approaches and the various reporters that can be generated as well as their utility.

Along with other groups, we have previously reported on safe harbors suitable for engineering in human ESC/iPSC lines^21^,^23^. The PPP1R12C/AAVS1 site is one of the best known genomic safe harbor (GSH) candidates in human genome^27^,^28^,^29^,^30^, while the other site, CLYBL/Chr13, has been reported as a safe harbor site^26^,^31^,^32^ though less studied. Further investigation will likely be needed for potential therapeutic applications^33^. Here we show that ubiquitous reporters (GFP or Luciferase driven by continuously expressed promoters) can be efficiently inserted into these two sites (PPP1R12C/AAVS1 and CLYBL/Chr13) by TALEN^34^. No reporters were silenced during directed differentiation or random differentiation in any of the safe harbor KI lines. Luciferase can be detected and quantified in media from as few as 10^4^ neurons or astrocytes differentiated from the KI lines in 96-well format, indicating the utility of such lines for high-throughput screening. In addition, we show that identical lines can be made in different allelic backgrounds, eliminating potential integration site effects.

The Nanoluc-HaloTag reporter construct offered several advantages. For example, it allows for simultaneous quantitative assessment and fluorescent imaging on demand for time-lapse imaging. By using small molecule ligands to HaloTag, one also has the advantage of choosing fluorescent signals, as its labeling is transient, which allows for other imaging modalities to be used when necessary. In addition, antibodies to the HaloTag are available allowing antibody labeling in fixed cells for archival purposes.

The limited availability of human cells of the central nervous system make iPSC derived neural derivatives a promising novel cell source for drug discovery and for improvement of existing drug development workflow, specifically for the evaluation of toxicity and efficacy of lead compounds. Most neural differentiation protocols currently available, however, produce a heterogeneous population of neuron and glial cells, making it difficult to interpret the mechanism of action of a given compound. Our neural lineage-specific KI reporter approach was designed to mitigate this problem, using a donor vector containing dual reporters of luciferase and HaloTag attached to the C-terminal of an endogenous lineage-specific gene. We chose to target MAP2 gene for neuron-specific reporter and GFAP gene for astrocyte-specific reporter, and validated lineage-specific expression of the reporters during lineage-specific differentiation. The MAP2-Nanoluc-KI line allows for real-time monitoring of neuronal differentiation, and the luciferase activity in the culture reflects the percentage of neurons. The non-disruptive assay format enables accurate and quantitative measurement of any compound on neurons specifically. Likewise, the GFAP-Nanoluc-KI line allows for tracking and quantitative measurement of astrocytes in culture. The fact that as few as 10^4^ cells (neurons or astrocytes) from these KI lines were needed to detect the luciferase activity in culture media makes our reporters/assays applicable for high-throughput and high content screening.

Although we did not find it necessary, it is important to note that because drug selection is incorporated via a T2A site downstream of the lineage specific promoter, it is possible to purify neurons and astrocytes for assays. Likewise, cells can be sorted using the fluorescent label allowing one to combine screening with gene expression analysis.

Our cassette exchange in iPSC and NSC show that clones can be rapidly generated in multiple stages of development. Although we tested only in the neuronal lineage there is no reason to suppose this will not work with any other intermediate progenitor. Indeed our preliminary results show that this works with mesenchymal stem cells and astrocyte precursors as well. The ability to use multiple reporters in the same site for different purposes is an invaluable benefit to having to generate new reporters or new lines and test their specificity and quality each time.

An additional advantage of our approach is that the same safe harbors, master and control lines can be used for other lineages. This would allow us to develop a database of drug responses or effects of a mutation in a single pathway in multiple cell types from a single allelic background. This has simply not been possible before and represents an advantage of iPSC-based screens when combined with reporter assays in a reference or control line. In summary, we provide a novel platform for the rapid generation of gene-targeted iPSC lines using a common donor vector, well-characterized site and a master line strategy. We believe that the availability of this toolbox and corresponding reagents will permit others to generate similar lines in different allelic backgrounds, and thus be of significant interest to the community, representing a novel application of genome editing.

## Methods

### iPSC culture and gene targeting by ZFN/TALENs

A subclone of each NCRM1 and NCRM5 integration-free iPSC line (NIH CRM), named XCL1 and XCL5, was obtained from XCell Science (Novato, CA) and used as the parental cells for all engineered work described in this study. iPSC were cultured as previously described^35^,^36^ and maintained in feeder-free conditions on Matrigel (BD Biosciences, CA) coated dishes using mTeSR™1 media (STEMCELL Technologies Inc., Vancouver, Canada) following the manufacturer's protocols.

TALEN expression plasmids targeting safe harbor sites in Chr.13 and Chr.19 (AAVS1) were provided by NIH. ZFN expression plasmids targeting the C-term of MAP2 and GFAP genes were purchased from Sigma (St. Louis, MO). Each plasmid DNA was linearized by XbaI for mRNA production and purification following modified manufacturer's protocols.

### Donor vector design and construction

A backbone vector containing a puromycin resistant gene flanked by two loxP sites and a CAG promoter driving copGFP cassette was constructed between the lox2272 and lox511 sites. Insulator expressing genes were used to generate AAVS1-copGFP donor vector targeting to the AAVS1 site at Chr.19 (Fig. 1a). A 754 bp left homologous arm and an 838 bp right homologous arm were amplified by PCR from XCL1 (Xcell Inc, CA) gDNA and cloned into the backbone vector. For Chr13-copGFP, a similar backbone vector was used (the puromycin resistant gene was replaced by a PGK promoter driving neomycin resistant gene) and inserted with a 832 bp left homologous arm and a 796 bp right homologous arm amplified from the Chr13 safe harbor region where designed TALENs are targeting to.

Another backbone vector containing a P2A peptide, Nanoluc reporter gene fused with a downstream HaloTag, a T2A peptide in frame with a Neomycin resistant gene and a puromycin resistant gene flanked by two loxP sites was designed and constructed for targeting different genes to generate lineage-specific reporter donor vectors. A 1069 bp left homologous arm right before the stop codon of MAP2 gene was PCRed from XCL1, and then cloned into the backbone vector upstream and in frame with the P2A peptide. A 1084 bp fragment was cloned in as right homologous arm to generate the MAP2-Nanoluc-KI donor vector. For the GFAP-Nanoluc-KI donor vector, a 1022 bp fragment right before the stop codon and a 1020 bp fragment after the stop codon was cloned into backbone vector as the left and right homologous arm, respectively.

### Reporter iPSC lines generation

Prior to nucleofection, XCL1 iPSC were maintained and passed using Accutase (Life Tech., NJ) to make sure cells are growing in monolayer. On the day of nucleofection, single cell suspension cells were generated using Accutase followed by inactivation and washes with HBSS. 4–6 μg of each pair of TALENs/ZFNs RNA was used for nucleofection using Amaxa Human Stem Cell Nucleofection Kit (Lonza, NJ). After nucleofection, cells were plated in mTeSR™1 medium with 10 μM Rock inhibitor. After 2–5 days recovery, cells were treated with appropriate antibiotics. Specifically, 2.5 μg/ml Puromycin (Life Tech., NJ) for AAVS1-copGFP, MAP2-nanoluc-KI and GFAP-Nanoluc-KI lines and 500 μg/ml Neomycin (Life Tech., NJ) for Chr13-copGFP line. Drug resistant colonies were re-plated at low density for single cell cloning. Colonies growing from single cells were screened by PCRs and sequencing to identify targets with correct donor vector integrations. The verified targets were expanded, stored and characterized for future experiments.

### NSC derivation and neural differentiation

Generation of NSC was accomplished as previously described^37^. In brief, NSC were derived from iPSC lines and were cultured on Matrigel coated dishes in Neurobasal® medium supplemented with 1% nonessential amino acids, 1% GlutaMAX, 1 x B-27®, and 10 ng/ml bFGF, and passaged using Accutase. Neuronal differentiation was achieved by culturing NSC in Neuronal Primer media (Xcell inc, CA) on a surface coated with Poly-L-ornithine (2 μg/ml, Sigma, St. Louis, MO) and laminin (10 μg/ml, Life Tech., NJ) at a density of 40-50 k/cm2 for 5-6 days until cells become confluent. Then cells were split with Accutase and were plated onto new poly-ornithine/laminin coated dishes at 40-50 k/cm2 in Neuronal Medium (Xcell inc, CA) to continue differentiation for as long as desired. Astrocyte differentiation from NSC was also carried out on culture dishes or glass cover slips coated with Poly-L-ornithine/laminin in Astrocyte Primer medium (Xcell Inc, CA). Medium was changed every other day and cells have to be split at least 3 times before day 15. On day 18, change media to Astrocyte medium (Xcell inc, CA) and continue differentiation for up to day 35.

### Cre recombinase-mediated cassette exchange in iPSC and NSC

The iPSC or NSC master lines (AAVS1-copGFP or Chr13-copGFP) were plated on Matrigel-coated 35 mm dishes. When cells reached 70–80% confluency, plasmid expressing Cre recombinase and daughter construct were co-transfected using Lipofectamine 3000 (Life Tech., NJ) following manufacturer's protocols. For iPSC, cells were selected with appropriate antibiotics to enrich the cell populations with successful cassette exchange. Then drug resistant colonies were further screened using a fluorescence microscope to identify colonies that lost green fluorescence, which were picked, expanded and confirmed by PCR and sequencing.

### Immunocytochemistry

Immunocytochemistry and staining procedures were as described previously^38^. Briefly, cells were fixed with 4% paraformaldehyde for 20 minutes at room temperature, blocked in blocking buffer (10% goat serum, 1% BSA, 0.1% Triton X-100) for one hour followed by incubation with the primary antibody at 4°C overnight in 8% goat serum, 1% BSA, 0.1% Triton X-100. Appropriately coupled secondary antibodies, Alexa488 and Alexa594 (Molecular Probes and Jackson ImmunoResearch Lab Inc.) were used for single or double labeling. All secondary antibodies were tested for cross reactivity and non-specific immune-reactivity. The following primary antibodies were used: Nestin (BD Biosciences, CA), GFAP (DakoCytomation Inc, CA), MAP2 (Sigma, St. Louis) and DCX (Santa Cruz Biotechnology, TX). DAPI was used to label the nuclei.

### Luciferase activity measurement and HaloTag detection

Determination of Nanoluc luciferase activity was measured using Nano-Glo Assay System following manufacturer's protocol (Promega, WI). In brief, 50 μl culture media was mixed with 50 μl of Nano-Glo Assay Reagent in a 96-well plate for an incubation period of 5 min. Then luciferase activity was measured using a Perkin Elmer Fusion-alpha-FP-HT universal microplate analyser. Detection of HaloTag was achieved either in live cells using HaloTag® TMR Ligand following manufacturer's protocol (Promega, WI) or in fixed cells using HaloTag antibodies (Promega, WI).

### qPCR analysis

Quantitative PCR reactions were carried out on the CFX96TM Touch Bio-Rad instrument (Bio-Rad, CA) using iTaqTM Universal SYBR® Green supermix (Bio-Rad, CA) according to the manufacturers' instructions. PCR reactions were conducted in six replicates for each sample. Human ACTB was amplified as internal standards for normalization. Primers specific for “copGFP” were used to amplify donor vector specific products. Amplifications from each samples were calculated using ΔΔCt method and normalized against endogenous ACTB. The copy number of donor vector in each testing line was presented as % mean control using “CAG-GFP-Chr19” line as the control.

### Fluorescence-activated cell sorting (FACS)

FACS was performed as described in (Liu et al 2012^39^). Briefly, NSCs were detached using Accutase and filtered through a 70μM nylon filter. BD FACSAria special order system and FACSDiva 6.1.1 software was used for sorting.

## Author Contributions

Y.P., M.S.R. and X.Z.: conception and experimental designs. Y.P., G.S., R.S. and A.S. conducted experiments. Y.P., M.R.S. and X.Z. wrote the manuscript and all authors reviewed the manuscript.

## Supplementary Material

Supplementary Information Supp Fig and table

## References

[b1] Mali P. *et al.* RNA-guided human genome engineering via Cas9. Science 339, 823-826; 10.1126/science.1232033 (2013). 23287722 PMC3712628

[b2] Boch J. *et al.* Breaking the code of DNA binding specificity of TAL-type III effectors. Science 326, 1509–1512; 10.1126/science.1178811 (2009). 19933107

[b3] Urnov F. D. *et al.* Highly efficient endogenous human gene correction using designed zinc-finger nucleases. Nature 435, 646–651; 10.1038/nature03556 (2005). 15806097

[b4] Moscou M. J. & Bogdanove A. J. A simple cipher governs DNA recognition by TAL effectors. Science 326, 1501; 10.1126/science.1178817 (2009). 19933106

[b5] Shaltouki A., Peng J., Liu Q., Rao M. S. & Zeng X. Efficient generation of astrocytes from human pluripotent stem cells in defined conditions. Stem Cells 31, 941–952; 10.1002/stem.1334 (2013). 23341249

[b6] Han Y. *et al.* Identification by automated screening of a small molecule that selectively eliminates neural stem cells derived from hESCs but not dopamine neurons. PloS one 4, e7155; 10.1371/journal.pone.0007155 (2009). 19774075 PMC2743191

[b7] Matsa E., Burridge P. W. & Wu J. C. Human stem cells for modeling heart disease and for drug discovery. Sci. Transl. Med. 6, 239ps236; 10.1126/scitranslmed.3008921 (2014). PMC4215696 24898747

[b8] Sinnecker D. *et al.* Modeling long-QT syndromes with iPS cells. J Cardiovasc Transl Res. 6, 31–36; 10.1007/s12265-012-9416-1 (2013). 23076501

[b9] Laustriat D., Gide J. & Peschanski M. Human pluripotent stem cells in drug discovery and predictive toxicology. Biochem. Soc. Trans. 38, 1051–1057; 10.1042/BST0381051 (2010). 20659002

[b10] Sinnecker D., Laugwitz K. L. & Moretti A. Induced pluripotent stem cell-derived cardiomyocytes for drug development and toxicity testing. Pharmacol. Ther. 143, 246–252; 10.1016/j.pharmthera.2014.03.004 (2014). 24657289

[b11] Shtrichman R., Germanguz I. & Itskovitz-Eldor J. Induced pluripotent stem cells (iPSCs) derived from different cell sources and their potential for regenerative and personalized medicine. Curr. Mol. Med. 13, 792–805 (2013). 23642060 10.2174/1566524011313050010

[b12] Kumar K. K., Aboud A. A. & Bowman A. B. The potential of induced pluripotent stem cells as a translational model for neurotoxicological risk. Neurotoxicology 33, 518-529; 10.1016/j.neuro.2012.02.005 (2012). 22330734 PMC3358591

[b13] Ananiev G., Williams E. C., Li H. & Chang Q. Isogenic pairs of wild type and mutant induced pluripotent stem cell (iPSC) lines from Rett syndrome patients as in vitro disease model. PLoS One 6, e25255; 10.1371/journal.pone.0025255 (2011). 21966470 PMC3180386

[b14] Vojnits K. & Bremer S. Challenges of using pluripotent stem cells for safety assessments of substances. Toxicology 270, 10–17; 10.1016/j.tox.2009.12.003 (2010). 20004228

[b15] Fu X. & Xu Y. Challenges to the clinical application of pluripotent stem cells: towards genomic and functional stability. Genome Med. 4, 55; 10.1186/gm354 (2012). 22741526 PMC3698533

[b16] Ho P. J., Yen M. L., Yet S. F. & Yen B. L. Current applications of human pluripotent stem cells: possibilities and challenges. Cell transplantation 21, 801–814; 10.3727/096368911X627507 (2012). 22449556

[b17] Sun X., Tan G. & Liew R. Future challenges for patient-specific induced pluripotent stem cells in cardiovascular medicine. Expert Rev. Cardiovasc. Ther. 10, 943–945; 10.1586/erc.12.88 (2012). 23030280

[b18] Tabar V. & Studer L. Pluripotent stem cells in regenerative medicine: challenges and recent progress. Nat. Rev. Genet. 15, 82–92; 10.1038/nrg3563 (2014). 24434846 PMC4539940

[b19] Wang J. *et al.* Targeted gene addition to a predetermined site in the human genome using a ZFN-based nicking enzyme. Genome Res. 22, 1316–1326; 10.1101/gr.122879.111 (2012). 22434427 PMC3396372

[b20] Holkers M. *et al.* Differential integrity of TALE nuclease genes following adenoviral and lentiviral vector gene transfer into human cells. Nucleic Acids Res. 41, e63; 10.1093/nar/gks1446 (2013). 23275534 PMC3597656

[b21] Luo Y. *et al.* Stable Enhanced Green Fluorescent Protein Expression After Differentiation and Transplantation of Reporter Human Induced Pluripotent Stem Cells Generated by AAVS1 Transcription Activator-Like Effector Nucleases. Stem Cells Transl. Med. 3, 821–835; 10.5966/sctm.2013-0212 (2014). 24833591 PMC4073825

[b22] Maggio I. *et al.* Adenoviral vector delivery of RNA-guided CRISPR/Cas9 nuclease complexes induces targeted mutagenesis in a diverse array of human cells. Sci. Rep. 4, 5105; 10.1038/srep05105 (2014). 24870050 PMC4037712

[b23] Macarthur C. C. *et al.* Chromatin insulator elements block transgene silencing in engineered human embryonic stem cell lines at a defined chromosome 13 locus. Stem Cells Dev. 21, 191–205; 10.1089/scd.2011.0163 (2012). 21699412 PMC3258440

[b24] Yan Y. *et al.* Efficient and rapid derivation of primitive neural stem cells and generation of brain subtype neurons from human pluripotent stem cells. Stem Cells Transl. Med. 2, 862–870; 10.5966/sctm.2013-0080 (2013). 24113065 PMC3808201

[b25] Swistowska A. M. *et al.* Stage-specific role for shh in dopaminergic differentiation of human embryonic stem cells induced by stromal cells. Stem Cells Dev. 19, 71–82; 10.1089/scd.2009.0107 (2010). 19788370

[b26] Cerbini T. *et al.* TALEN-mediated CLYBL targeting enables enhanced transgene expression and one-step generation of multiplexed reporter human iPSC and NSC lines. PloS one In Press (2014). 10.1371/journal.pone.0116032 PMC4294658 25587899

[b27] Luo Y., Rao M. & Zou J. Generation of GFP Reporter Human Induced Pluripotent Stem Cells Using AAVS1 Safe Harbor Transcription Activator-Like Effector Nuclease. Curr. Protoc. Stem Cell Biol. 29, 5A 7 1-5A 7 18; 10.1002/9780470151808.sc05a07s29 (2014). PMC4128243 24838915

[b28] Tiyaboonchai A. *et al.* Utilization of the AAVS1 safe harbor locus for hematopoietic specific transgene expression and gene knockdown in human ES cells. Stem cell Res. 12, 630–637; 10.1016/j.scr.2014.02.004 (2014). 24631742 PMC4048956

[b29] Owens R. A. Second generation adeno-associated virus type 2-based gene therapy systems with the potential for preferential integration into AAVS1. Curr. Gene Ther. 2, 145–159 (2002). 12109212 10.2174/1566523024605627

[b30] Recchia A. & Mavilio F. Site-specific integration by the adeno-associated virus rep protein. Curr. Gene Ther. 11, 399–405 (2011). 21827397 10.2174/156652311797415809

[b31] Thyagarajan B. *et al.* Creation of engineered human embryonic stem cell lines using phiC31 integrase. Stem cells 26, 119–126; 10.1634/stemcells.2007-0283 (2008). 17962703

[b32] Liu Y. *et al.* Generation of platform human embryonic stem cell lines that allow efficient targeting at a predetermined genomic location. Stem Cells Dev. 18, 1459–1472; 10.1089/scd.2009.0047 (2009). 19355838

[b33] Sadelain M., Papapetrou E. P. & Bushman F. D. Safe harbours for the integration of new DNA in the human genome. Nat. Rev. Cancer 12, 51–58; 10.1038/nrc3179 (2012). 22129804

[b34] Zhu H., Lensch M. W., Cahan P. & Daley G. Q. Investigating monogenic and complex diseases with pluripotent stem cells. Nat. Rev. Genet. 12, 266–275; 10.1038/nrg2951 (2011). 21386866

[b35] Lie K. H., Chung H. C. & Sidhu K. S. Derivation, propagation, and characterization of neuroprogenitors from pluripotent stem cells (hESCs and hiPSCs). Methods Mol. Biol. 873, 237–246; 10.1007/978-1-61779-794-1_15 (2012). 22528359

[b36] Zou J. *et al.* Oxidase-deficient neutrophils from X-linked chronic granulomatous disease iPS cells: functional correction by zinc finger nuclease-mediated safe harbor targeting. Blood 117, 5561–5572; 10.1182/blood-2010-12-328161 (2011). 21411759 PMC3110021

[b37] Swistowski A. *et al.* Xeno-free defined conditions for culture of human embryonic stem cells, neural stem cells and dopaminergic neurons derived from them. PloS one 4, e6233; 10.1371/journal.pone.0006233 (2009). 19597550 PMC2705186

[b38] Zeng X. *et al.* Stable expression of hrGFP by mouse embryonic stem cells: promoter activity in the undifferentiated state and during dopaminergic neural differentiation. Stem Cells 21, 647–653; 10.1634/stemcells.21-6-647 (2003). 14595124

[b39] Liu Q. *et al.* Human neural crest stem cells derived from human ESCs and induced pluripotent stem cells: induction, maintenance, and differentiation into functional schwann cells. Stem Cells Transl. Med. 1, 266–278; 10.5966/sctm.2011-0042 (2012). 23197806 PMC3659695

