# Supplementary material for: A platform for rapid generation of single and multiplexed reporters in human iPSC lines
Source: Sci Rep. 2015 Mar 17;5:9205. doi: 10.1038/srep09205 (PMC4361878; doi:10.1038/srep09205)
Supplement: Supplementary Information — Supp Fig and table [file srep09205-s1.pdf]

A platform for rapid generation of single and multiplexed reporters in human iPSC lines

Ying Pei[1], Guadalupe Sierra[2], Renuka Sivapatham[1], Andrzej Swistowski[2], Mahendra S Rao[3,],
and Xianmin Zeng[1.2]

[1]Buck Institute for Age Research, Novato, CA

[2]XCell Science, Novato, CA

[3]NxCell Inc, Novato, CA

Running title: iPSC reporters

Correspondence: Xianmin Zeng, Ph.D. Buck Institute for Research on Aging, Novato, CA
94945, USA; Tel: 415-209-2211; E-mail: xzeng@buckinstitute.org

Supplementary figure legends

Sup Fig. 1. Genotyping and karyotyping for the master line. (a) Normal karyotype analysis was observed for AAVS1-copGFP iPS cell line. (b) Three-germ layer staining with AFP, SMA and β III-tublin markers showed that the copGFP in the AAVS1-copGFP line was not silenced after random differentiation. Random differentiation was performed as previously described[27]. (c) The Chr13-Nanoluc line in NCRM5 (XCL5) background was used to test whether luciferase reporter is silenced during neural differentiation. The bar graph demonstrated that the luciferase signals were constitutively active in the Chr13-Nanoluc line after differentiation into either neurons or astrocytes.

Sup Fig. 2. Validation of pluripotency and genomic stability of the KI lines. (a) MAP2-Nanoluc-KI iPSC showed normal expression of pluripotency markers Oct4, Tra 1-60, Nanog and Tra 1-81. (b) Normal karyotype analysis was observed for MAP2-Nanoluc-KI iPSC line. (c) GFAP-Nanoluc-KI iPSC showed normal expression of pluripotency markers Oct4, Tra 1-60, Nanog and Tra1-81. (d) Normal karyotype analysis was observed for GFAP-Nanoluc-KI iPSC line.

Supp. Fig. 1

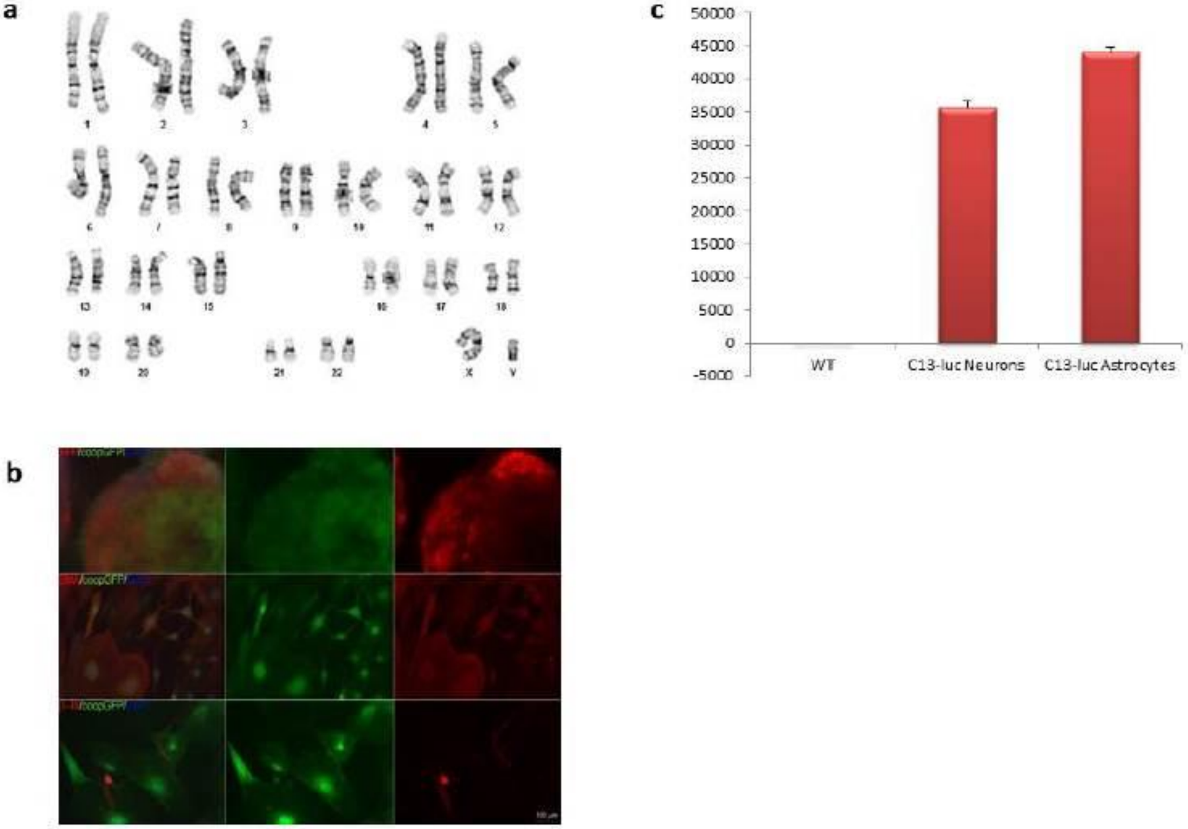
a
b
c
50000
45000
40000
35000
30000
25000
20000
15000
10000
5000
0
-5000
WT
C13-luc Neurons
C13-luc Astrocytes

Supp. Fig. 2

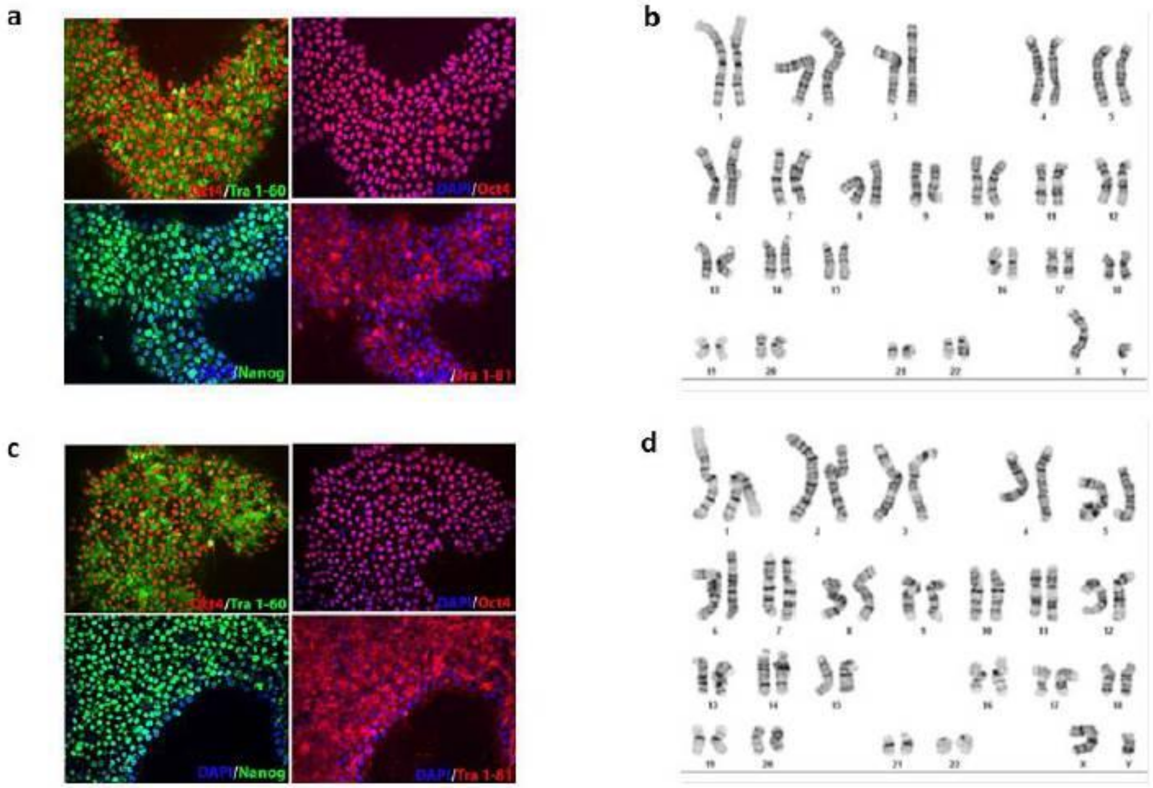

Supplementary Table 1. Expression levels of genes adjacent to MAP2 and GFAP

SYMBOL	XCL1 neuron ctrl	MAP2 neuron KI
**Genes upstream of MAP2**		
CASP10	14.8	2.7
CASP8	-12.4	-15.2
CD28	-8.4	-10.7
CREB1	4983.4	3205.0
CRYGD	-9.6	-8.9
CTLA4	-1.4	-8.7
ICOS	27.3	24.7
IDH1	2314.5	4767.3
NRP2	-10.0	-13.9
SUMO1	70.6	180.4
**Genes downstream of MAP2**		
CXCR1	-1.8	-3.9
CYP27A1	137.6	35.1
DES	-0.4	-4.5
ERBB4	12.0	-5.6
FN1	-7.9	-2.1
IGFBP2	5107.1	2862.9
IHH	1.3	9.5
SLC11A1	-9.2	-21.8

SYMBOL	XCL1 astrocyte ctrl	GFAP astrocyte KI
**Genes upstream of GFAP**		
ADAM11	13.2	-8.9
ASB16	-18.7	-17.0
CCDC43	434.6	361.5
DBF4B	6.7	-18.4
EFTUD2	1921.9	1702.0
FZD2	638.1	629.9
GJC1	10596.8	5648.2
GPATCH8	49.9	42.1
GRN	972.5	418.9
HDAC5	26.0	23.3
HIGD1B	-5.1	-9.7
ITGA2B	-5.6	7.1
SLC4A1	-10.4	-20.7
UBTF	99.9	93.7
**Genes downstream of GFAP**		
ARHGAP27	-9.3	-1.7

CBX1	1297.1	1149.9
CRHR1	5.9	9.6
FMNL1	-7.0	22.1
HEXIM1	149.2	81.5
HEXIM2	75.7	57.2
KIF18B	-14.1	1.3
MAP3K14	70.3	36.2
MAPT	104.3	7.2
MGC57346	232.8	109.0
NMT1	373.0	200.9
PLCD3	635.2	327.7
TBX21	221.6	199.6
